# Supplementary material for: Effects of radial radio-frequency field inhomogeneity on MAS solid-state NMR experiments
Source: Magn Reson (Gott). 2021 Jul 1;2(1):523–43. doi: 10.5194/mr-2-523-2021 (PMC10539735; doi:10.5194/mr-2-523-2021)
Supplement: The supplement related to this article is available online at: https://doi.org/10.5194/mr-2-523-2021-supplement. [file mr-2-523-supplement.pdf]

Supplement of Magn. Reson., 2, 523–543, 2021
https://doi.org/10.5194/mr-2-523-2021-supplement
© Author(s) 2021. CC BY 4.0 License.

Open Access
MAGNETIC
RESONANCE

Supplement of

Effects of radial radio-frequency field inhomogeneity on MAS solid-state NMR experiments

Kathrin Aebischer et al.

Correspondence to: Matthias Ernst (maer@ethz.ch)

The copyright of individual parts of the supplement might differ from the article licence.

S1 Second-order scaling factors

Expressions for second-order scaling factors for $\chi = y$

$$q_y^{(n_1,n_2)} = \sum_{\nu,\lambda} \frac{1}{\nu\omega_\mathrm{m} + \lambda\omega_\mathrm{eff}} \left(a_z^{(-\nu-c\cdot n_1,-\lambda)} a_x^{(\nu-c\cdot n_2,\lambda)} - a_x^{(-\nu-c\cdot n_1,-\lambda)} a_z^{(\nu-c\cdot n_2,\lambda)} \right), \tag{S1}$$

$$q_{y,\mu}^{(n_1,n_2)} = \sum_{\nu,\lambda} \frac{1}{\nu\omega_\mathrm{m} + \lambda\omega_\mathrm{eff}} \Big(a_z^{(-\nu-c\cdot n_1,-\lambda)} a_{x\mu}^{(\nu-c\cdot n_2,\lambda)} - a_x^{(-\nu-c\cdot n_1,-\lambda)} a_{z\mu}^{(\nu-c\cdot n_2,\lambda)} - a_z^{(\nu-c\cdot n_1,\lambda)} a_{x\mu}^{(-\nu-c\cdot n_2,-\lambda)} + a_x^{(\nu-c\cdot n_1,\lambda)} a_{z\mu}^{(-\nu-c\cdot n_2,-\lambda)} \Big), \tag{S2}$$

$$p_y^{(n_1,n_2)} = \sum_{\mu} \sum_{\nu,\lambda} \frac{1}{\nu\omega_\mathrm{m} + \lambda\omega_\mathrm{eff}} \left(a_{z\mu}^{(-\nu-c\cdot n_1,-\lambda)} a_{x\mu}^{(\nu-c\cdot n_2,\lambda)} - a_{x\mu}^{(-\nu-c\cdot n_1,-\lambda)} a_{z\mu}^{(\nu-c\cdot n_2,\lambda)} \right), \tag{S3}$$

$$p_{\mu y\xi}^{(n_1,n_2)} = \sum_{\nu,\lambda} \frac{1}{\nu\omega_\mathrm{m} + \lambda\omega_\mathrm{eff}} \Big(a_{\mu z}^{(-\nu-c\cdot n_1,-\lambda)} a_{x\xi}^{(\nu-c\cdot n_2,\lambda)} - a_{\mu z}^{(\nu-c\cdot n_1,\lambda)} a_{x\xi}^{(-\nu-c\cdot n_2,-\lambda)} - a_{\mu x}^{(-\nu-c\cdot n_1,-\lambda)} a_{z\xi}^{(\nu-c\cdot n_2,\lambda)} + a_{\mu x}^{(\nu-c\cdot n_1,\lambda)} a_{z\xi}^{(-\nu-c\cdot n_2,-\lambda)} \Big). \tag{S4}$$

Expressions for second-order scaling factors for $\chi = z$

$$q_z^{(n_1,n_2)} = \sum_{\nu,\lambda} \frac{1}{\nu\omega_\mathrm{m} + \lambda\omega_\mathrm{eff}} \left(a_x^{(-\nu-c\cdot n_1,-\lambda)} a_y^{(\nu-c\cdot n_2,\lambda)} - a_y^{(-\nu-c\cdot n_1,-\lambda)} a_x^{(\nu-c\cdot n_2,\lambda)} \right), \tag{S5}$$

$$q_{z,\mu}^{(n_1,n_2)} = \sum_{\nu,\lambda} \frac{1}{\nu\omega_\mathrm{m} + \lambda\omega_\mathrm{eff}} \Big(a_x^{(-\nu-c\cdot n_1,-\lambda)} a_{y\mu}^{(\nu-c\cdot n_2,\lambda)} - a_y^{(-\nu-c\cdot n_1,-\lambda)} a_{x\mu}^{(\nu-c\cdot n_2,\lambda)} - a_x^{(\nu-c\cdot n_1,\lambda)} a_{y\mu}^{(-\nu-c\cdot n_2,-\lambda)} + a_y^{(\nu-c\cdot n_1,\lambda)} a_{x\mu}^{(-\nu-c\cdot n_2,-\lambda)} \Big), \tag{S6}$$

$$p_z^{(n_1,n_2)} = \sum_{\mu} \sum_{\nu,\lambda} \frac{1}{\nu\omega_\mathrm{m} + \lambda\omega_\mathrm{eff}} \left(a_{x\mu}^{(-\nu-c\cdot n_1,-\lambda)} a_{y\mu}^{(\nu-c\cdot n_2,\lambda)} - a_{y\mu}^{(-\nu-c\cdot n_1,-\lambda)} a_{x\mu}^{(\nu-c\cdot n_2,\lambda)} \right), \tag{S7}$$

$$p_{\mu z\xi}^{(n_1,n_2)} = \sum_{\nu,\lambda} \frac{1}{\nu\omega_\mathrm{m} + \lambda\omega_\mathrm{eff}} \Big(a_{\mu x}^{(-\nu-c\cdot n_1,-\lambda)} a_{y\xi}^{(\nu-c\cdot n_2,\lambda)} - a_{\mu x}^{(\nu-c\cdot n_1,\lambda)} a_{y\xi}^{(-\nu-c\cdot n_2,-\lambda)} - a_{\mu y}^{(-\nu-c\cdot n_1,-\lambda)} a_{x\xi}^{(\nu-c\cdot n_2,\lambda)} + a_{\mu y}^{(\nu-c\cdot n_1,\lambda)} a_{x\xi}^{(-\nu-c\cdot n_2,-\lambda)} \Big). \tag{S8}$$

S2 Simulation Parameters

Table S1. Parameters used in numerical simulations of the MAS experiments presented in the main text. The number of crystallite orientations used for the ZCW (Cheng et al., 1973) powder averaging is denoted by N_p. Unless noted otherwise, all simulations were performed at an external static magnetic field of $B_0 = 14.1$ T, corresponding to a proton resonance frequency of 600 MHz.

Experiment	Figure	Probe	MAS [kHz]	N_p	Spin	$\omega_{1,\mathrm{nom}}$ [kHz]
Nutation	3a	3.2 mm	30	1	H	100
	3b	3.2 mm	15	1	H	100
	3c	1.3 mm	30	1	H	100
	3d	1.3 mm	15	1	H	100
	S1	3.2 mm	30	1	H	100
	S2b	3.2 mm	15	100	HH	100
R3	S3a & c	3.2 mm	10	538	IS	10
CP	6	3.2 mm	20	1154	CN	^{13}C: 85 ; ^{15}N: 65
	6	3.2 mm	20	1154	HN	^{1}H: 70 ; ^{15}N: 50
	6	3.2 mm	20	1154	HC	^{1}H: 90 ; ^{13}C: 70
tmSPICE CP [1]	7	3.2 mm	20	1154	NC	^{15}N: 40 ; ^{13}C: 40
REDOR	8a	1.3 mm	20	538	CN	^{13}C: 100 ; ^{15}N: 62.5
	8b & S6a	3.2 mm	20	538	CN	^{13}C: 62.5 ; ^{15}N: 50
	8c	1.9 mm	40	10000	HN	^{1}H: 125 ; ^{15}N: 50
	8d	1.9 mm	40	10000	HN	^{1}H: 125 ; ^{15}N: 50
C7 and PC7	9a-d	3.2 mm	10	538	CC	70
	9e-f [2]	3.2 mm	10	538	CC	70
FSLG	10 & S11	3.2 mm	12.5	1154	HHH	125 (along θ_m)
	S7	3.2 mm	ca. 14.1	1154	HHH	125 (along θ_m)

[1] Simulations at a B_0-field of 9.4 T, corresponding to a proton resonance frequency of 400 MHz.
[2] Simulations at a B_0-field of 7.0 T, corresponding to a proton resonance frequency of 300 MHz.

S2.1 Spin System Parameters

Table S2. Input parameters for CSA tensors used in tmSPICE CP simulations of NCA and NCO polarization transfers at a proton resonance frequency of 400 MHz. Spin 0 corresponds to ^{15}N, spin 1 corresponds to ^{13}C. Euler angles α, β, γ are given in degree.

Spin System	Spin	$a_{\text{iso}}^{\text{CSA}}$ [Hz]	δ^{CSA} [Hz]	η^{CSA}	Relative tensor orientation (α, β, γ)
NCA	0	0.0	3960.0	0.19	(103.01, -141.57, 65.13)
	1	0.0	-2000.0	0.43	(-81.06, 37.80, 37.44)
NCO	0	0.0	3960.0	0.19	(103.01, -141.57, 65.13)
	1	0.0	-7600.0	0.90	(100.87, -127.26, -131.30)

Table S3. Scalar J-couplings J_{IS}, anisotropies δ_{IS} and the orientation of the dipolar coupling tensors used in simulations of tmSPICE NCA and NCO polarization transfers at a proton resonance frequency of 400 MHz. Euler angles α, β, γ are given in degree.

Spin pair	J_{IS} [Hz]	$\frac{\delta_{IS}}{2\pi}$ [Hz]	Relative tensor orientation (α, β, γ)
NCA (0, 1)	-11	2038.34	(0.0, 63.76, 113.83)
NCO (0, 1)	-15	2698.98	(0.0, 72.69, -27.58)

Table S4. Input parameters for CSA tensors used in C7 and PC7 simulations of a homonuclear CC two-spin system based on phthalic acid (Hellwagner et al., 2017) at a carbon resonance frequency of 75 MHz. Euler angles α, β, γ are given in degree.

Spin	$a_{\text{iso}}^{\text{CSA}}$ [Hz]	δ^{CSA} [Hz]	η^{CSA}	Relative tensor orientation (α, β, γ)
0	-300	-5775	0.69	(90, 70, 10)
1	300	5775	0.69	(90, 110, 70)

Table S5. Input parameters for CSA tensors used in FSLG simulations of a dipolar coupled three proton spin system at a resonance frequency of 600 MHz. Parameters are inspired by glycine. Euler angles α, β, γ are given in degree.

Spin	$a_{\text{iso}}^{\text{CSA}}$ [Hz]	δ^{CSA} [Hz]	η^{CSA}	Relative tensor orientation (α, β, γ)
0	1932	2100	0.80	(0, 30.59, 147.04)
1	2508	-2974.8	0.751	(-54.76, 109.2, 96.18)
2	4752	2085.6	0.998	(-17.82, 103.4, 78.42)

Table S6. Anisotropy δ_{IS} and the relative orientation of the dipolar coupling tensors used in FSLG simulations of a three proton spin system at a resonance frequency of 600 MHz. Scalar J-couplings were set to zero. Parameters are inspired by glycine. Euler angles α, β, γ are given in degree.

Spin pair	$\frac{\delta_{IS}}{2\pi}$ [Hz]	Relative tensor orientation (α, β, γ)
(0, 1)	-52245	(0, 70.77 , 120.47)
(0, 2)	-13595	(134.37, 96.59, 14.88)
(1, 2)	-13387	(-105.82, 85.98, 47.20)

S3 Nutation Spectra

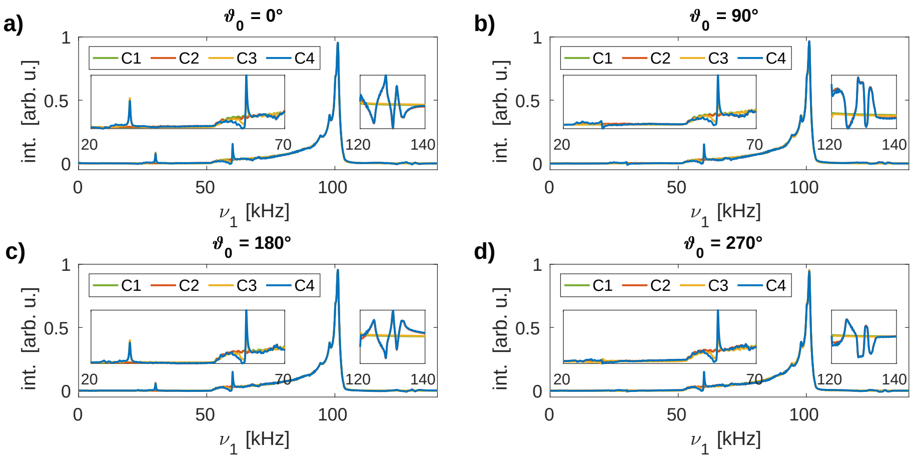

Figure S1. Simulated nutation spectra at a resonance frequency of 600 MHz for the 3.2 mm MAS probe assuming a spinning frequency of 30 kHz and a nominal rf amplitude of 100 kHz. Shown are spectra of rz-planes with initial ϑ_0 values of 0° (a), 90° (b), 180° (c) and 270° (d). The initial orientation only influences the phases of the sidebands arising due to the MAS modulation of the rf amplitude and phase.

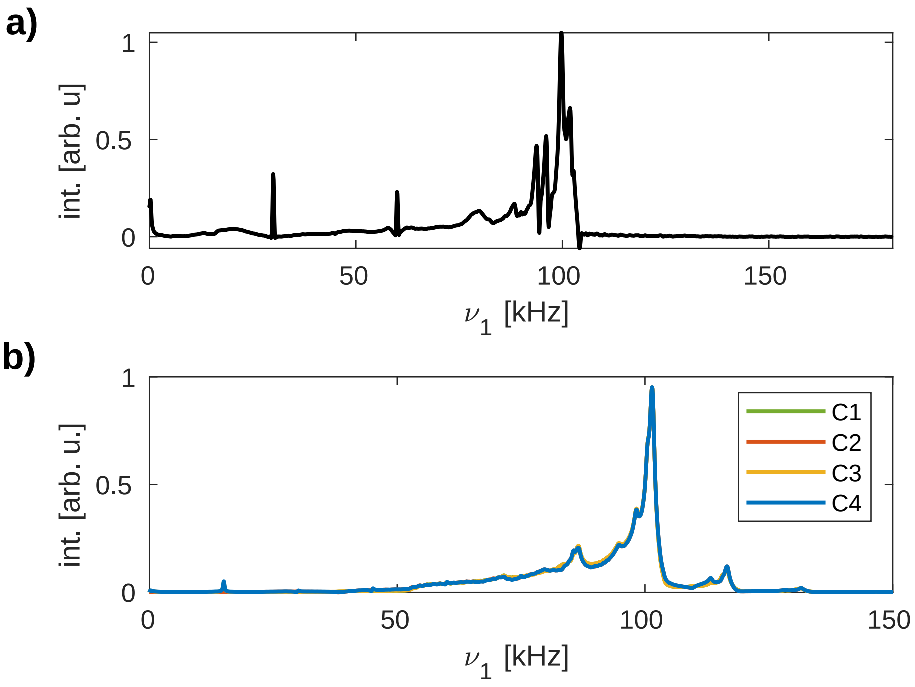

Figure S2. a) Experimental ^{1}H nutation spectrum of natural-abundance adamantane recorded at a proton resonance frequency of 500 MHz in a Bruker 1.9 mm MAS probe spinning at 30 kHz. The nominal rf amplitude was set to 100 kHz as calibrated using a nutation spectrum. As was seen in the experimental nutation spectra of glycine in Fig. 4 in the main text, sidebands due to MAS modulation of the rf phase arise at 30 and 60 kHz. However, no sideband is observed at 130 kHz which is consistent with the observations made in simulated nutation spectra of single-spin systems (s. Fig. 3 in the main text). b) Simulated nutation spectrum of a dipolar coupled two-spin system ($\frac{\delta_{IS}}{2\pi} = 50$ kHz, scalar J-couplings and chemical shifts set to zero) at a resonance frequency of 600 MHz for the 3.2 mm MAS probe assuming a spinning frequency of 15 kHz and a nominal rf amplitude of 100 kHz for C1–C4. In comparison to the simulated single-spin spectra (s. Fig. 3 in the main text), significantly stronger sidebands at $\nu_1 \pm n \cdot \nu_\mathrm{r}$ arise for all four cases. The shape of this sideband replicates the main nutation profile. This agrees with the observations made in the experimental nutation spectra of glycine shown in Fig. 4 in the main text.

S4 Rotary Resonance Recoupling

Rotary resonance recoupling was simulated in a heteronuclear two-spin system with an anisotropy of the dipolar coupling of $\frac{\delta_{IS}}{2\pi}$ = -1.9 kHz at a spinning frequency of 10 kHz. The spinlock with a nominal rf amplitude of 10 kHz (thus $\nu_\mathrm{r} = \nu_1$) was applied on the non-observed S spin and the expectation value of the $\hat{I}_x$ operator detected as a function of time. 538 crystallite orientations were used for the powder averaging. Resulting FIDs and spectra are shown in Fig. S3 for simulations in the 3.2 mm probe (Fig. S3a and c) and for simulations assuming an analytical sine-modulation of the rf amplitude and phase (Fig. S3b and d, where $\omega_{1,\mathrm{rel}}(t) = 1 + 0.05 \cdot \sin(\omega_\mathrm{r} t)$ and $\phi_\mathrm{rel}(t) = 0.05 \cdot \sin(\omega_\mathrm{r} t)$). An additional linebroadening of 50 Hz was applied during processing.

As described in Levitt et al. (1988), an additional central feature is observed when rf phase modulations are present (C3 and C4 in Fig. S3d), since such phase modulations lead to a decay of magnetization during the spinlock. In these spectra, this additional peak is quite strong since a rather large amplitude of 5% was assumed for the sine-modulation of the rf phase. In simulated spectra for the 3.2 mm on the other hand, such a central feature is observed for both C1 (only average rf amplitude offsets) and C4 (both rf amplitude and phase modulated due to the radial rf inhomogeneity). In C1, the additional peak arises due to the fact that the spinlock amplitude experienced in parts of the sample will not satisfy the rotary resonance recoupling condition due to the static rf amplitude offset. Thus, spins that are not recoupled will contribute to the signal and the FID will not oscillate around zero (s. Fig. S3a). The rf phase modulations that are present in the simulations of C4 now lead to a broadening of this central feature due to the decay of magnetization during the spinlock.

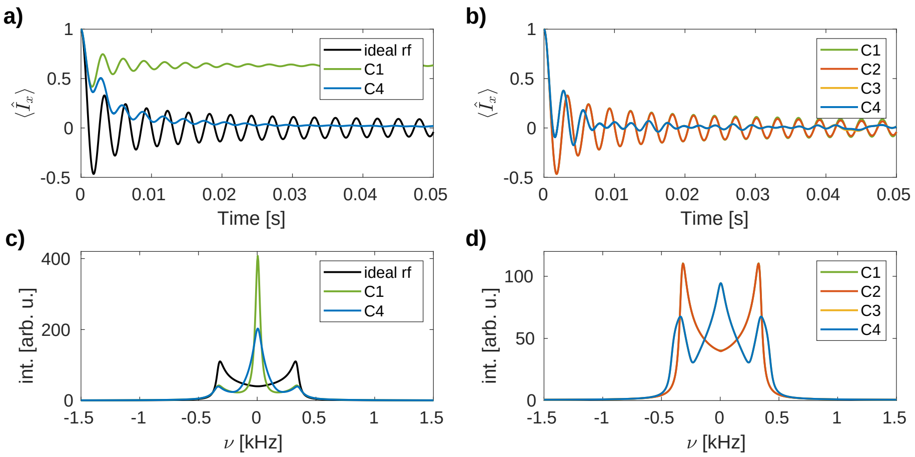

Figure S3. Simulated FIDs (a and b) and corresponding spectra (c and d) of a heteronuclear two-spin system with a $\frac{\delta_{IS}}{2\pi}$ = -1.9 kHz under rotary resonance recoupling assuming a MAS frequency of 10 kHz and a nominal rf amplitude of 10 kHz (spinlock is applied on the non-observed spin). a)/c) Simulation results for the 3.2 mm probe. Shown are C1 (only average rf amplitude offsets) and C4 (both amplitude and phase modulations taken into account). The resulting FID and spectrum assuming a perfectly homogeneous rf field are shown as black solid lines. b)/d) Simulation results for C1-C4 assuming a sine-shaped modulation of the rf amplitude and phase with an amplitude of 5% ($\omega_{1,\mathrm{rel}}(t) = 1 + 0.05 \cdot \sin(\omega_\mathrm{r} t)$ and $\phi_\mathrm{rel}(t) = 0.05 \cdot \sin(\omega_\mathrm{r} t)$).

S5 Cross Polarization

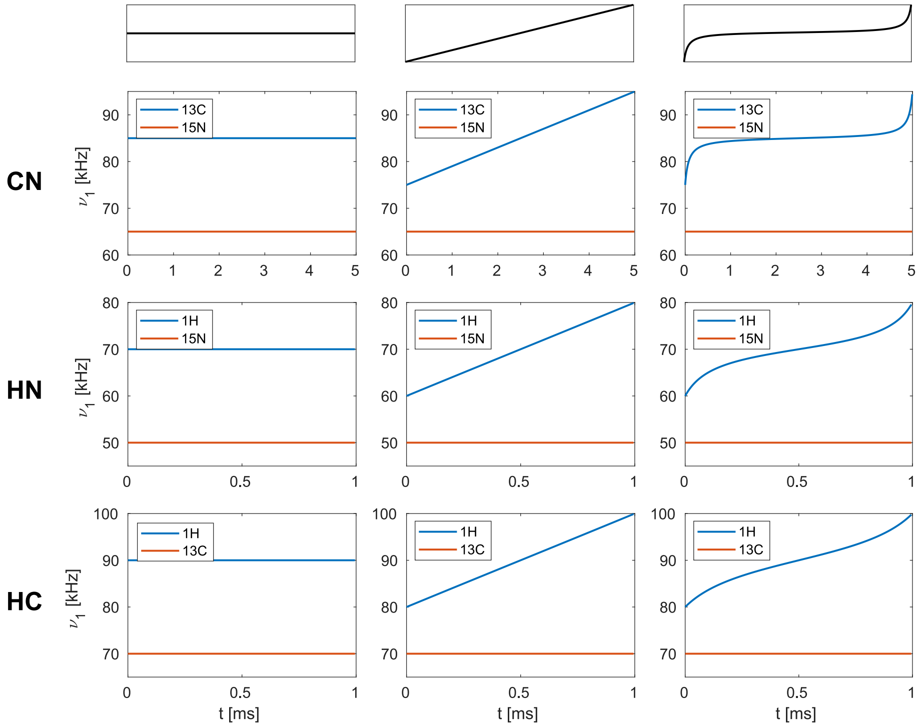

Figure S4. Nominal rf-field amplitude modulations for simulated CP polarization transfers in CN (top row), HN (middle row) and HC (bottom row) spin pairs. Transfers were simulated assuming a MAS frequency of 20 kHz.

In the numerical simulations of adiabatic passage CP, the shape of the amplitude modulation during the contact time τ on one of the channels was chosen to be of the following form

$$\chi^{\Delta}(t) = \mid \omega_{\mathrm{IS}}^{\mathrm{est}} \mid \tan(\alpha \cdot \left[\frac{\tau}{2} - t\right]), \tag{S9}$$

where the time-dependent mismatch χ^{Δ} is defined as $\chi^{\Delta}(t) = \omega_{1\mathrm{I}}(t) - \omega_{1\mathrm{S}} - n\omega_{\mathrm{r}}$ and the parameter α given by

$$\alpha = \frac{2}{\tau} \arctan\left(\frac{\chi_i^{\Delta}}{\mid \omega_{\mathrm{IS}}^{\mathrm{est}} \mid}\right). \tag{S10}$$

χ_i^{Δ} corresponds to the initial offset from the matching condition and was set to -10 kHz in all simulations. The steepness is determined by the estimated dipolar coupling constant $\mid \omega_{\mathrm{IS}}^{\mathrm{est}} \mid$. The nominal rf amplitude on the other channel was kept constant during the contact time. Estimated anisotropies of the dipolar coupling $\frac{\delta_{IS}}{2\pi}$ of 475, 3125 and 5750 Hz were used for CN, HN and HC spin pairs respectively.

S6 REDOR

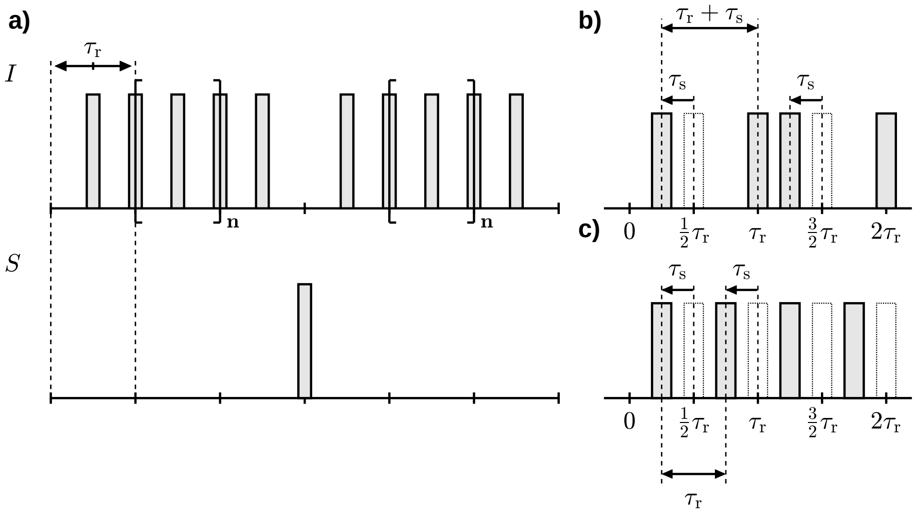

Figure S5. a) Schematic representation of the pulse sequence for the standard REDOR implementation. In the basic building block, the two π pulses are separated by half a rotor period τ_r. Signal detection occurs on the S-spin channel after integer multiples of the cycle time. b) REDOR implementation where the π pulse at $\frac{\tau_r}{2}$ is shifted in time by τ_s (Gullion and Schaefer, 1989b). c) REDOR implementation where both π pulses in the basic building block are shifted. The time interval between consecutive pulses is kept constant at $\frac{\tau_r}{2}$ (Jain et al., 2019). In both implementations, pulses are shifted in a mirror symmetric way during the second half of the pulse sequence.

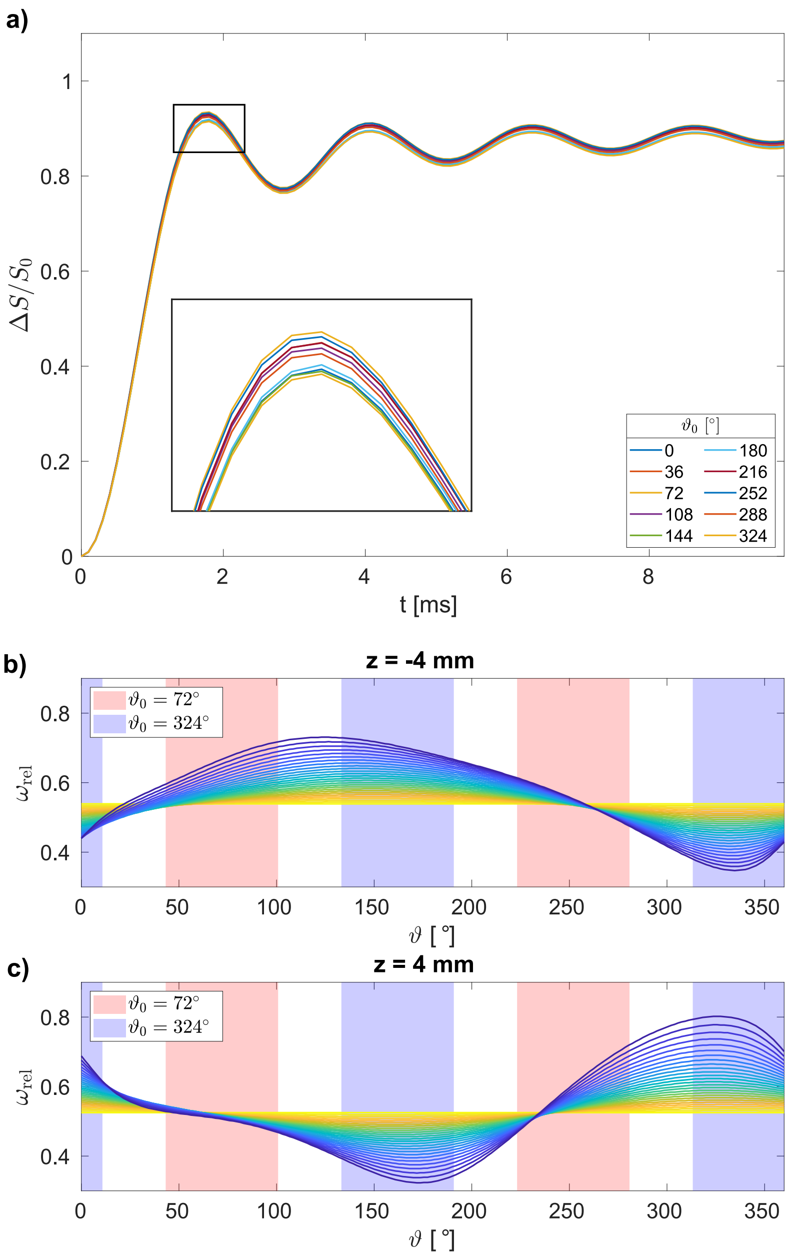

Figure S6. a) Simulated REDOR curves (C4 only) for a CN spin pair (anisotropy of the dipolar coupling of $\frac{\delta_{IS}}{2\pi} = 2$ kHz) for rz-planes at different ϑ_0 in a 3.2 mm MAS probe. Simulation parameters are the same as for the results shown in Fig. 8b in the main text. Slight differences in the recoupling efficiencies are observed depending on the initial ϑ_0 value. However, these differences are only marginal. b) / c) Rf amplitude trajectories as a function of ϑ for $z =$ -4 (b) and 4 mm (c) for all 27 r values (from $r_{\min} = 0$ mm in yellow to $r_{\max} = 1.3$ mm in blue). The positions of the π pulses for rz-planes which showed maximum ($\vartheta_0 = 72^\circ$) and minimum ($\vartheta_0 = 324^\circ$) recoupling efficiency (a) are indicated by the areas shaded in red and blue. The slightly higher recoupling efficiency observed for $\vartheta_0 = 72^\circ$ observed can be explained by the timing of pulses such that they occur during intervals where the rf amplitude does not experience large rf amplitude modulations.

S7 Frequency-Switched Lee-Goldburg

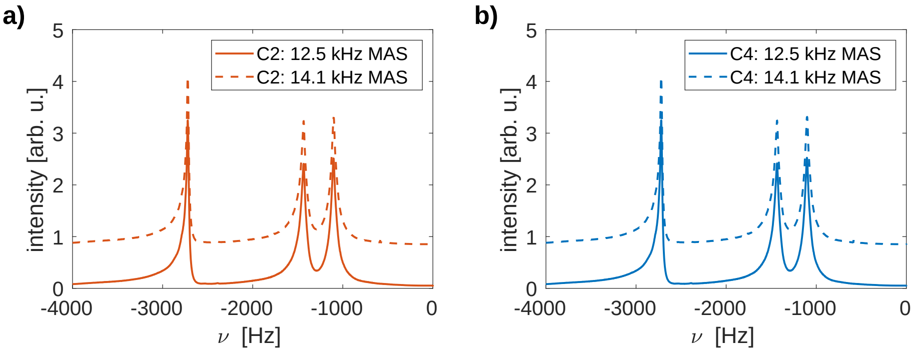

Figure S7. Simulated FSLG decoupled homonuclear spectra of a three-spin system at a resonance frequency of 600 MHz for the 3.2 mm MAS probe. The effective field strength along the magic angle was set to 125 kHz. Shown are cases where rf amplitude modulations were taken into account (C2 in a, C4 in b). In these cases broadening was observed in the simulated spectra shown in the main text in Fig. 10. In addition to the synchronous implementation of the FSLG decoupling assuming a MAS frequency of 12.5 kHz an asynchronous implementation assuming a MAS frequency of approximately 14.1 kHz is shown (with a y-offset for better visualization). The same broadening is observed for both implementations and can thus be attributed to the rf amplitude modulations due to the radial rf inhomogeneity instead of potential resonant effects.

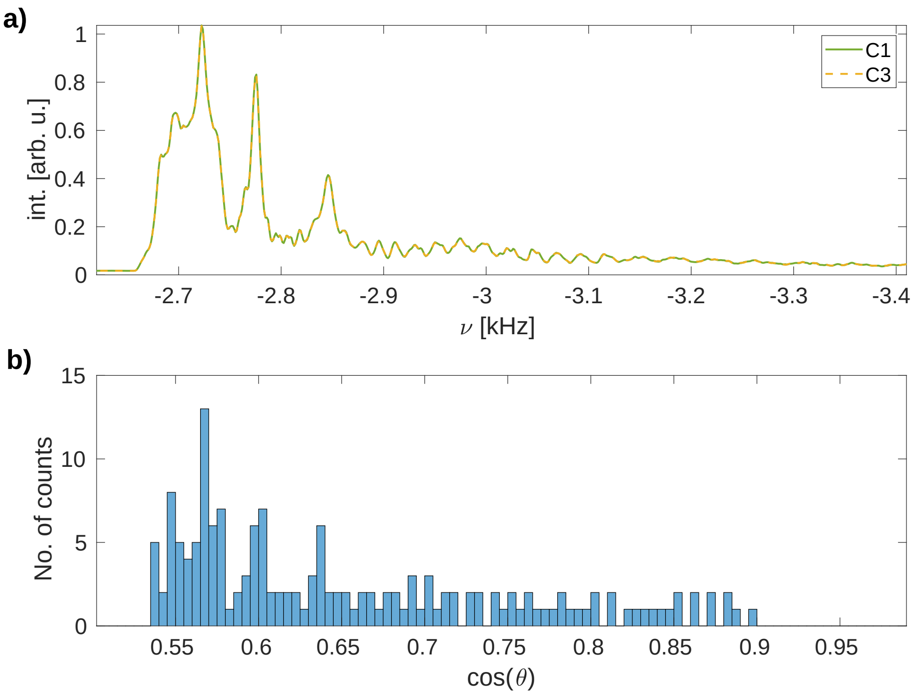

Figure S8. a) Detail of the observed line splitting for C1 and C3 in the contribution of the radial slice at $r = 1.3$ mm to the simulated FSLG decoupled spectra as shown in Fig. 10c in the main text. b) Histogram of the distribution of the isotropic chemical-shift scaling factors $\cos(\theta)$ (with θ being the angle between the effective field and the z-axis computed as $\sin(\theta) = \frac{\nu_{1,\mathrm{nom}} \cdot \bar{\nu}_{1,\mathrm{rel}}}{\nu_{\mathrm{eff}}}$ for each volume element in this radial slice) for the radial slice at $r = 1.3$ mm. The observed splitting is in good agreement with this distribution.

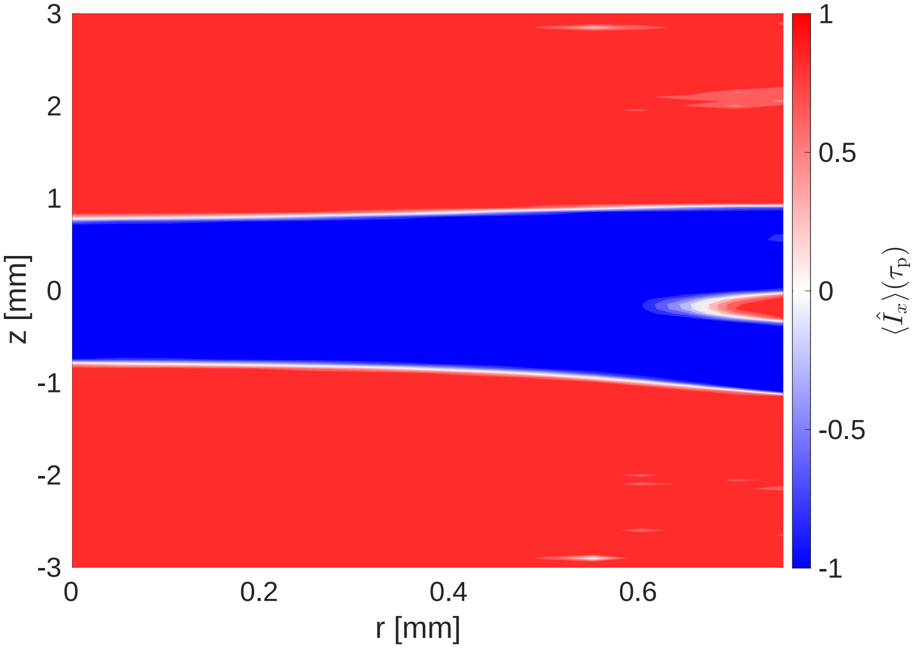

Figure S9. Simulated inversion profile of a $2\ \mathrm{ms}$ I-BURP-2 pulse in the spin-lock frame in a $1.9\ \mathrm{mm}$ MAS probe assuming a spinning speed of $14\ \mathrm{kHz}$. The nominal rf-field amplitude was set to $100\ \mathrm{kHz}$ which also corresponds to the modulation frequency of the nutation-frequency selective pulse. Shown is the expectation value of the $\hat{I}_x$ spin operator at the end of the pulse (the initial density operator was set to $\hat{I}_x$). One can see that the central area of the rotor, where the rf amplitude corresponds to the nominal rf amplitude, is selectively inverted by the pulse.

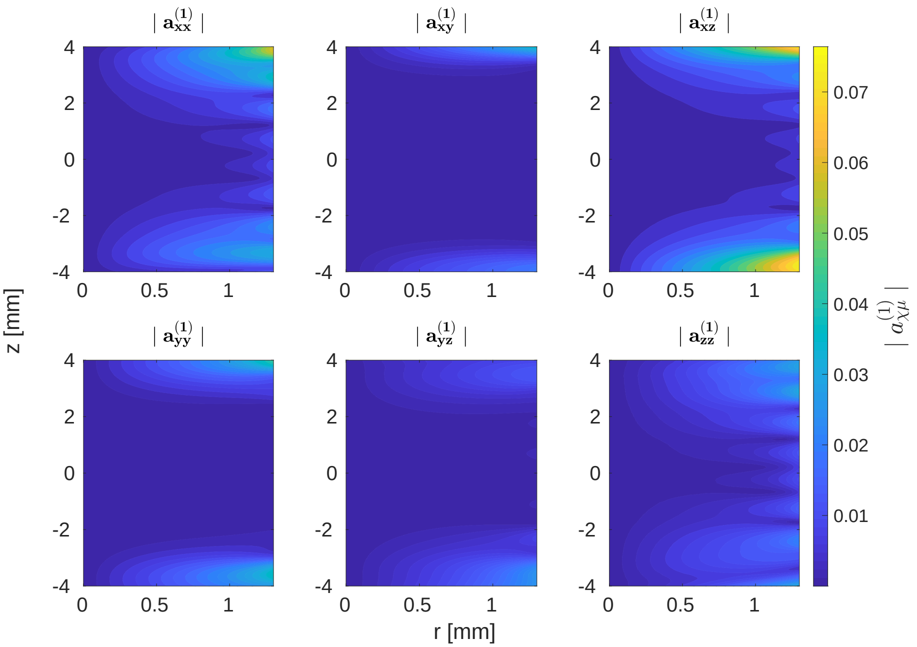

Figure S10. Scaling factors of the homonuclear dipolar coupling terms $a_{\chi\mu}^{(k=1)}$ in the first-order effective Hamiltonian during FSLG decoupling as a function of the position within the sample space for a 3.2 mm MAS probe for C4. Coefficients were extracted from interaction-frame trajectories of single spin operators for a nominal rf-field strength of 125 kHz along the magic angle assuming a MAS frequency of 12.5 kHz. As the same interaction-frame trajectory was assumed for all spins, Fourier coefficients do not depend on the order of χ and μ ($a_{\chi\mu}^{(k)} = a_{\mu\chi}^{(k)}$). The individual $a_{\chi\mu}^{(k=1)}$ contributions to the norm shown in Fig. 13 in the main text are plotted separately to give insight into the two-spin terms that are reintroduced due to time-dependent rf modulations.

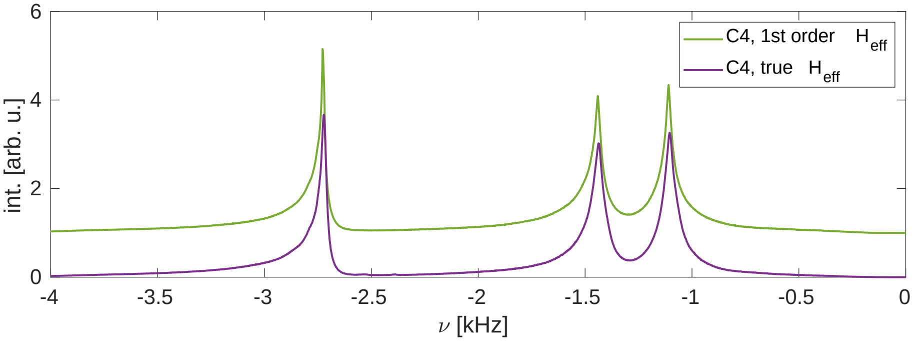

Figure S11. Comparison of simulated spectra of a three-spin system under FSLG decoupling (600 MHz proton resonance frequency, 12.5 kHz MAS, 125 kHz effective field along θ_m, 3.2 mm probe, see Tables S5 and S6 for spin system details) for C4 under the first-order effective Hamiltonian (green, s. Eq. (27) in the main text, scaling factors $a_\chi^{(k,l)}$ and $a_{\chi\mu}^{(k,l)}$ were computed numerically (s. Fig. 13 in the main text)) and the true effective Hamiltonian (purple). The simulated spectrum for the first-order effective Hamiltonian is shown with a y-offset of +1 for better visualization. The true effective Hamiltonian was back-calculated from the propagator over a full rotor cycle $\hat{U}(\tau_\mathrm{r})$ (determined by time-slicing of the Hamiltonian) as $\hat{\mathcal{H}}_\mathrm{eff}^{(\mathrm{true})} = \frac{\ln(\hat{U}(\tau_\mathrm{r}))}{i2\pi\tau_\mathrm{r}}$. An additional linebroadening of 1 Hz was applied during processing. The obtained spectra for the true and the first-order effective Hamiltonians are very similar, indicating that the linewidth is dominated by first-order effects and the observed broadening in simulated spectra for C4 (s. Fig. 10 in the main text) can indeed be attributed to the reintroduction of homonuclear dipolar coupling terms to first order.

References

Levitt, M. H., Oas, T. G., and Griffin, R. G.: Rotary Resonance Recoupling in Heteronuclear Spin Pair Systems, Israel J. Chem., 28, 271–282, 1988.
